# Pathogenesis of central nervous system germ cell tumors

**DOI:** 10.3389/fonc.2022.991484

**Published:** 2022-09-09

**Authors:** Siyuan Liu, Linan Ren, Xue Gao, Mengjin Hao, Guixia Wang

**Affiliations:** Department of Endocrinology and Metabolism, The First Hospital of Jilin University, Changchun, China

**Keywords:** chromosomal deformity, gene mutation, hypomethylation of DNA, immune abnormalities, intracranial tumor

## Abstract

Intracranial germ cell tumors (IGCTs) are clinically rare. They are more common in children and adolescents and the incidence in Asia is higher than in Western countries. Histologically, IGCTs are divided into germinoma and non-germinomatous germ cell tumor (NGGCT). Germinoma is sensitive to radiotherapy and chemotherapy and therefore, patients with germinoma have a good prognosis. However, NGGCTs, especially those with malignant components, are not sensitive to radiotherapy and chemoradiotherapy, leading to a poor prognosis. The pathogenesis of IGCTs is not fully understood. By summarizing previous literature, we found that the occurrence of IGCTs may be related to the following factors: chromosomal instability, MAPK and/or PI3K pathway changes, and DNA hypomethylation in pure germ cell tumors.

## Introduction

Intracranial germ cell tumors (IGCTs) mainly occur in the midline region, with the pineal gland being the most common site (50%), followed by the suprasellar region (about 20%–30%). Tumors outside the midline are often referred to as ectopic germ cell tumors and are mainly found in the basal ganglia and thalamus.

IGCTs are more common in children and adolescents, and the ratio of the incidence among males to the incidence among females is about 3-4:1 (1). In addition, the incidence in different geographical locations varies; the incidence in Asia reaches 8%-14%, several times of that in the West (0.5%–3%) (2). The difference in incidence among populations may be related to genes (3). Studies have shown that GCTs in the basal ganglia region are more common in Japan, and bifocal GCTs with synchronous lesions in the pineal gland and suprasellar region are more common in European and American areas (3). The variation of JMJD1C (A chromatin modification gene) is more pronounced among Japanese (4), which indirectly reflects the incidence difference among populations. The results need to be validated in a much larger cohort.

Histologically, IGCTs can be classified into eight types according to 2021 WHO guidelines (5), namely, mature teratoma, immature teratoma, teratoma with somatic-type malignancy, germinoma, embryonal carcinoma, yolk sac tumor, choriocarcinoma, and mixed germ cell tumor (GCT). Germinoma is generally referred to as pure germinoma, while the other seven types are collectively referred to as non-germinomatous germ cell tumor (NGGCT). Germinoma accounts for 65% - 75% of IGCTs and more than half of NGGCTs are mixed IGCTs (6, 7). The prognosis of different histological types of IGCTs varies. NGGCTs, except benign mature teratoma, are classified as non-germinomatous malignant germ cell tumors (NGMGCTs) due to their higher degree of malignancy and treatment resistance than germinoma.

Currently, the origin of IGCTs is still controversial. It is generally believed that IGCTs originate from primordial germ cells (PGCs) (8). During 4–6 weeks of gestation, dislocation of PGCs occurs during migration due to altered expression of additional mediators, leading to IGCTs. However, Sano et al. (9) proposed the “embryonic cell theory” and regarded that pluripotent embryonic cells migrated to produce IGCTs during the early development stage prior to the progression of PGCs. That is, GCTs are derived from primitive germ cells, whereas other NGGCT cells are not derived from primitive germ cells and should be considered as embryonic dysplasia with envelope cell origin. In addition, Chris Tan et al. (10) concluded that IGCTs might be developed from neural stem cells by upregulating the genes through OCT-4 promoter demethylation. The pathogenesis of ICGTs is also unclear. Bhattacharjee *et al.* (11),Rickert et al. (12) and Schneider et al. (13) found chromosomal abnormalities. Fukushima et al. (14) reported that MAPK pathway activation promoted the development of IGCTs, especially pure germinoma. Wang et al. (4) found novel somatic alterations in the AKT/mTOR pathway. Schulte et al. (15) examined global hypomethylation in ICGTs, which may be related to chromosome instability.

Although chemoradiotherapy is effective for most IGCTs, the side effects will degrade the long-term quality of life of patients and lead to a high recurrence rate. Therefore, it is extremely important to understand the pathogenesis of IGCTs and find new therapeutic targets.

## Chromosomal instability

From the perspective of molecular genetics, chromosome instability is common in IGCTs (14), especially in NGGCTs, with 12p gain (about 50%) being the most common phenomenon. It seems that 12p gain plays an important role in the occurrence and development of GCTs. Satomi K et al. (16) regarded that 12p gain may predict the prognosis and found a higher prevalence of 12p gain in NGGCTs than in germinoma. In addition, gains of X, 21q, 1q and 8q, and loss of fragments of 11q and 13q were found in IGCTs (4, 13, 14). However, previous studies have reported that the gain of 12p is not very common, while 21q gain and X total gain are the main chromosomal changes in IGCTs (14). Bhattacharjee et al. (11) also showed that the gain of 21q is a unique feature of IGCTs, which makes them different from other intracranial tumors and provides a theoretical basis for their inheritance.

## Activation of signaling pathways

**Figure 1:**
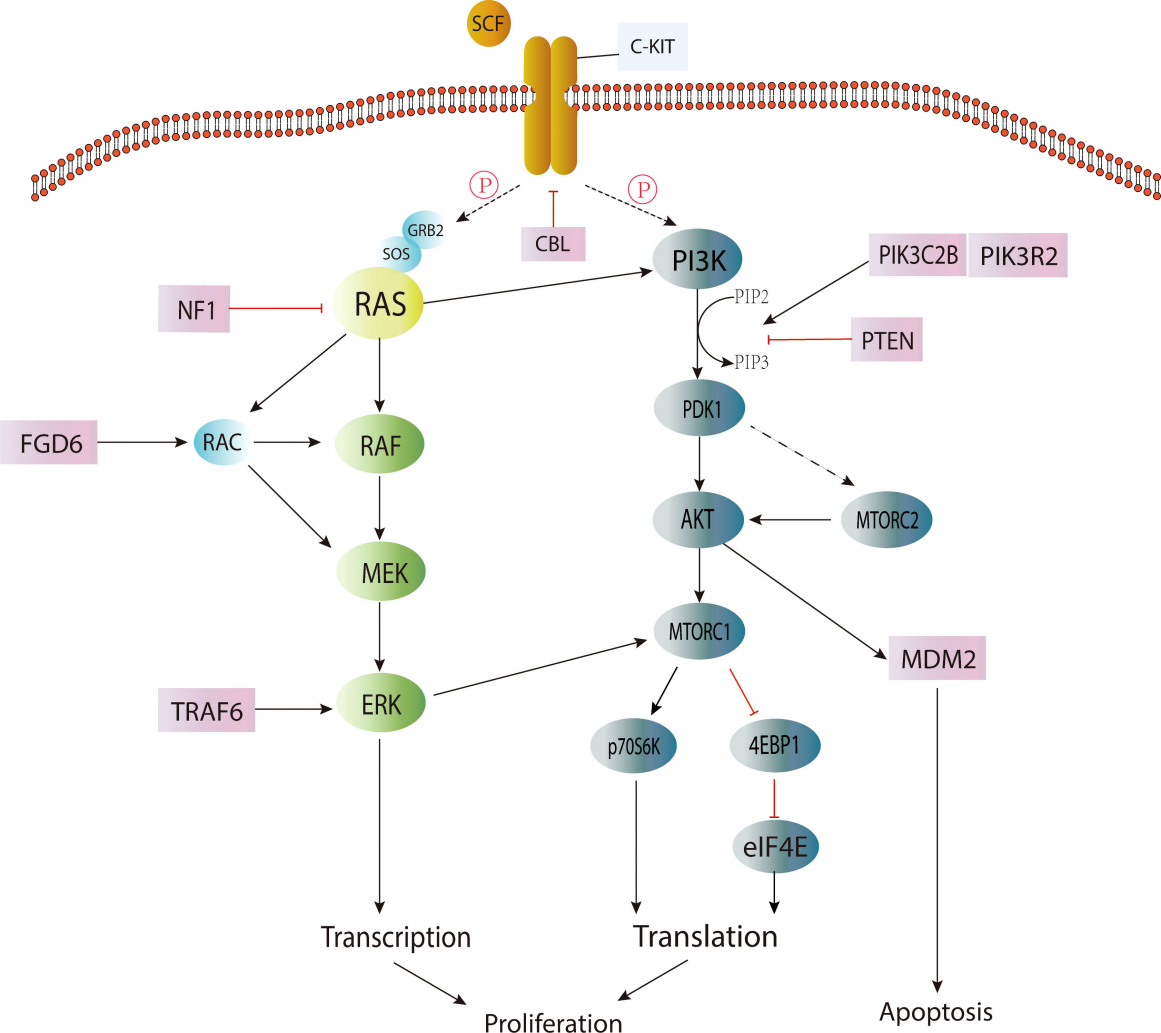
Signaling pathways involved in IGCT.

Activation of the KIT/RAS (MAPK) pathway and AKT/mTOR (PI3K) pathway by somatic point mutation is the genetic driver of most IGCTs. Changes in the MAPK pathway play a central role in the occurrence and development of IGCTs, and the mutation frequency of this pathway is higher in germinoma (14, 17). The mutation of genes in the PI3K pathway appears to be the second most common change in IGCTs, and the mutation frequency is similar between pure germinoma and NGGCTs (17). These mutations are also closely related to chromosomal instability (15) (**Figure 1**).

In the MAPK signaling pathway, the occurrence of IGCTs is closely related to the mutation acquisition function of the *KIT* gene. *KIT* (18) is a proto-oncogene encoding a transmembrane tyrosine kinase receptor, and its mutation will cause the permanent pathological activation of transmembrane protein without the binding of its ligand stem cell factor (SCF) (19), and then lead to the continuous proliferation of cells and the occurrence of tumors. It was found that the mutation hotspots were mainly located in the perimembrane region (exon 11) and the second tyrosine kinase domain (exon 17) (17). The expression of the KIT protein was detected in germinoma and more than half of NGGCTs (14), suggesting that upregulation of the MAPK pathway plays a central role in most IGCTs. However, *KIT* expression was also found in more than half of IGCTs without mutations in the MAPK/PI3K pathway. Therefore, whether the MAPK or PI3K pathways are activated by mechanisms or point mutations needs to be investigated (17).

The mutation of the *RAS* gene (20) is the second most common change in the MAPK pathway (20). RAS protein belongs to the GTPase family and is a regulator of cell proliferation, migration, apoptosis and survival. *RAS* gene mutation makes RAS protein maintain a continuously active GTP-binding state, which will lead to the development of tumors. Among the mutations of *RAS* genes, *KRAS* and *NRAS* mutations are the most common (4). However, it is worth noting that mutually exclusive *KIT/RAS* mutations are very common in pure germinoma (over 60%) but not common in NGGCTs (4, 14), and mixed GCTs with germinoma components account for the overwhelming majority of NGGCTs with *KIT/RAS* mutations (14), indicating that the mutation frequency of the MAPK pathway was higher in germinoma.

Other mutated genes involved in the MAPK pathway include *NF1*, *CBL*, *FGD6*, *FGFR2* and *TRAF6* (17), among which, *CBL* (21), as a tumor suppressor, refers to E3 ubiquitin ligase with RING finger domain. It can change the signal transduction pathway and suppress negatively regulated activation of receptor tyrosine kinases, including *KIT*, by regulating the ubiquitination of related proteins through E3 ligase. Studies (17) have found that *CBL* mutations mainly occur in the RING-type zinc finger domain, which can lead to the extension of the RTK signaling pathway. Therefore, the MAPK pathway may be upregulated, leading to the occurrence of tumors.

Genes in the PI3K pathway are the second most common alteration targets of CNS GCT (15, 17), such as *mTOR*, *PTEN*, *PIK3C2B*, and *PIK3R2* (17). The most common mutated gene in this pathway is *mTOR*, also known as the mammalian target of rapamycin. *MTOR* is an atypical serine/threonine kinase that interacts with a variety of proteins to form two distinct complexes, namely mTOR complex 1 (mTORC1) and mTOR complex 2 (mTORC2) (22).

Although the mutational hot-spots of mTOR are less obvious, about half of the mutations were found within the PI3/4 kinase domain (17). It was found that mTOR mutations promoted the phosphorylation of substrates p70S6K and 4EBP1 in nutrient deficiency, while mTORC2 promoted the expression of AKT and the phosphorylation of its target gene GSK3B and then activated the AKT pathway (17). AKT (23) is a silk/threonine protein kinase and a kind of downstream effector of PIP3K (phosphatidylinoside-3-kinase). When activated, it can promote cellular metabolism (e.g., glycogen, fatty acid and protein synthesis) and proliferation (24, 25). Somatic mutation in the AKT/mTOR (PI3K) pathway, especially frequent AKT1 amplification and mTOR mutation, may provide a basis for the treatment of IGCTs with AKT1/mTOR inhibitors (4). Recurrent mutations have been found in genes such as *BCORL1*, *TP53*, *SPTA1*, *KDM2A* and *LAMA4* (4). However, the pathogenesis of IGCTs without the above-mentioned pathway changes remains unclear. In addition, the exact pathogenesis of different subtypes of IGCTs remains to be studied (15). The WNT/β-catenin pathway may be associated with embryonic carcinoma (26), but the pathogenesis of other subtypes and mixed GCTs is currently unknown.

Mutations in genes may be related to gender and tumor location (27). A study reported that mutations in the MAPK pathway were found in about half of male cases but were rare in women, suggesting that the onset of the disease may be related to the activation of the MAPK pathway by the Y chromosome. In addition, recurrent mutations in BCORL1 (28) (a transcription corepressor and tumor suppressor located on the X chromosome) are associated with increased androgen levels during puberty in males with IGCTs and may account for the ICGT incidence difference across genders. In addition, studies showed that PI3K/mTOR pathway mutations and chromosome loss are more common in basal ganglia cases, but the specific mechanism was not clarified.

## DNA hypomethylation

In terms of epigenetics, IGCTs, especially germinoma, are characterized by DNA hypomethylation. Germinoma showed global DNA hypomethylation (15), a unique feature closely related to chromosome instability, but NGGCTs did not show this feature (29). In mixed cases, the germinoma component showed hypomethylation, while the non-germinoma component showed significantly higher methylation (19). Studies have found that the LINE1 methylation level of pure germinoma is significantly lower than that of normal tissues (19), which is attributed to DNA hypomethylation. LINE1 is the only active and autonomous retrotransposon in humans whose transposon activity is regulated by DNA methylation. Therefore, the LINE1 methylation level reflects the methylation status of the whole genome (30). However, the mechanism of LINE1 hypomethylation in pure germinoma remains to be studied. PGCs showed epigenetic characteristics of overall hypomethylation accompanied by high C-KIT expression, indicating that pure GCTs may originate from PGCs (29, 31). This phenomenon also suggests that there may be fundamental differences in cell origin or cell line differentiation between pure germinoma and NGGCTs.

## Immune abnormalities

IGCTs are also characterized by a high expression of immune response-related genes, such as CCL18, CD72, and IL6R, and a high level of lymphocytic infiltration (32). Programmed Death ligand 1 (PD-L1) is overexpressed in most GCTs (33). Moreover, tumor-infiltrating lymphocytes (TILs) frequently expresses programmed cell death receptor 1 (PD-1) (33). As an immune checkpoint, the PD-1/PD-L1 axis can weaken the anti-tumor immune system (34) and lead to subsequent tumor growth, suggesting that immune checkpoint inhibitors may be effective in the treatment of refractory germinoma infiltrated by a large number of immune cells (35).

In general, IGCTs are associated with chromosomal malformation, activation of the MAPK/PIK3 pathway, DNA hypomethylation, and the expression of immune-related genes. However, there are differences between germinoma and NGCGT. Previous transcriptome studies of IGCTs have shown that germinoma is characterized by the expression of genes involved in self-renewal (such as OCT4, NANOG, and KLF4) and immune response, while NGGCTs are characterized by the expression of genes involved in neuronal differentiation, WNT/β-catenin pathway, invasion, and epithelial-mesenchymal transformation (including SNAI2 (SLUG) and TWIST2) (36). In addition, a recent study on IGCTs from the genome-wide and transcriptome perspective showed that the expression profile of germinoma was similar to that of primitive germ cells, while the profile of IGCTs was similar to that of embryonic stem cells (37), which seems to confirm the hypothesis that the sources between pure germinoma and NGGCTs are inconsistent mentioned above. However, it is worth noting that changes in the MAPK/PIK3 pathway were found in both germinoma and NGGCTs, and the mutation frequency of the PI3K pathway in them was similar (17). Mixed cases with germinoma components and other components also showed common genetic mutations and different methylation characteristics (37), suggesting that the pathogenesis of germinoma and NGGCTs may overlap; that is, they may develop from the same origin cell by acquiring the same type of mutation and then differentiate through mechanisms other than genetic alterations, such as epigenetic modifications. Since this phenomenon cannot be explained by the theory of different-origin cells, researchers considered that the origin of IGCTs may be a common multipotent progenitor cell with MAPK/PI3K mutation that can generate PGCs or other differentiated cells through differential methylation and develop into germinoma or NGGCTs.

Researchers also regarded that the mutation of PGCs is the common ancestor of IGCTs. Mutated PGCs may directly develop into pure germinoma or differentiate into other types of IGCTs through epigenetic reprogramming.This hypothesis is verified by the finding that PGCs dedifferentiate into pluripotent stem cells and develop into teratoma in mice (38). Additionally, from the perspective of the tumor microenvironment, NGGCTs showed more abundant immune cell components, such as activated natural killer cells, monocytes and M2 macrophages, than pure GCTs (37).

Such mononuclear macrophage abundance was also reported in an independent IGCT cohort, and monocyte abundance in preoperative CSF was significantly associated with NGGCTs (6). This immunosuppressive phenotype of NGGCTs is important from an immunotherapy perspective, as it may be one of the potential causes of the treatment resistance of NGGCTs and its determination can promote the development of immunotherapies (37).

## Conclusion

IGCTs are clinically rare. Most of them are sensitive to radiotherapy and chemotherapy, and the 5-year overall survival rate (OSR) is more than 90% (39). However, the sensitivity of NGGCTs to chemoradiotherapy is low, and the 5-year OSR is less than 60% (40). Moreover, recurrence and long-term complications such as neurocognitive dysfunction and endocrine system diseases after treatment degrade the quality of life of patients. Therefore, it is particularly important to study the pathogenesis of IGCTs to identify new therapeutic targets. Since the occurrence of IGCTs is closely related to the mutation of the *KIT* gene, selective tyrosine kinase inhibitors targeting KIT are expected to be effective molecular targeting agents. Erk1/2 inhibitors (41) can inhibit MAPK signaling and tumor cell proliferation and therefore are expected to be effective for the treatment of IGCTs with *RAS* gene mutation. The mTOR inhibitor PP242 has been shown to down-regulate the AKT/mTOR pathway in a dose-dependent manner (17). LAE002, a small molecule ubiquitin-Akt kinase inhibitor, has shown effectiveness in various clinical trials (42). In addition, immunotherapy shows promising prospects. The high expression of PD-L1 in GCT cells and the high expression of PD-1 in TILs make the application of immune checkpoint inhibitors feasible. Moreover, the immunosuppressive phenotype of NGGCTs may contribute to reducing drug resistance. In conclusion, blocking the signaling pathways that induce IGCTs from the perspective of molecular mechanism and improving the tumor microenvironment from the perspective of immune mechanism provide new treatment ideas for IGCTs.

## Author contributions

Conceptualization- SL and LR. Writing- original draft preparation SL. Writing- review and editing LR, GW, and SL. Supervision- GW. Funding acquisition- GW. All authors read and approved the final version for submission.

## Conflict of interest

The authors declare that the research was conducted in the absence of any commercial or financial relationships that could be construed as a potential conflict of interest.

## Publisher’s note

All claims expressed in this article are solely those of the authors and do not necessarily represent those of their affiliated organizations, or those of the publisher, the editors and the reviewers. Any product that may be evaluated in this article, or claim that may be made by its manufacturer, is not guaranteed or endorsed by the publisher.
